# CX3CL1/CX3CR1 axis attenuates early brain injury via promoting the delivery of exosomal microRNA-124 from neuron to microglia after subarachnoid hemorrhage

**DOI:** 10.1186/s12974-020-01882-6

**Published:** 2020-07-14

**Authors:** Xiao Chen, Ming Jiang, Haiying Li, Yang Wang, Haitao Shen, Xiang Li, Yunhai Zhang, Jiang Wu, Zhengquan Yu, Gang Chen

**Affiliations:** 1 Department of Neurosurgery & Brain and Nerve Research Laboratory, The First Affiliated Hospital of Soochow University, 188 Shizi Street, Suzhou, 215006 China; 2 Department of Neurosurgery, The First Affiliated Hospital of University of Science and Technology of China, 17 Lujiang Road, Hefei, 230001 China; 3 Jiangsu Key Laboratory of Medical Optics, Suzhou Institute of Biomedical Engineering and Technology, Chinese Academy of Sciences, Suzhou, 215163 China

**Keywords:** Subarachnoid hemorrhage, Early brain injury, CX3CL1, CX3CR1, Microglia, Neuroinflammation, MicroRNA-124, Exosomes

## Abstract

**Background:**

Microglial activation-mediated neuroinflammation is a major contributor to early brain injury (EBI) after subarachnoid hemorrhage (SAH). MicroRNA-124 (miR-124) is the most abundant miRNAs in the central nervous system (CNS) and plays a vital role in microglial activation by targeting protein CCAAT-enhancer-binding protein α (C/EBPα). It has been reported that the CX3CL1/CX3CR1 axis is involved in the delivery of miR-124 from neurons to microglia.

**Methods:**

An experimental rat SAH model was established by injecting autologous arterial blood into the prechiasmatic cistern, and cultured primary neurons and microglia were exposed to oxyhemoglobin to mimic SAH in vitro. We additionally exploited specific expression plasmids encoding CX3CL1 and CX3CR1.

**Results:**

We observed significant decreases in CX3CL1 and CX3CR1 in the brain tissues of SAH patients. We also observed decreases in the levels of CX3CL1 in neurons and CX3CR1 in microglia after SAH in rats. Moreover, microglia exhibited an activated phenotype with macrophage-like morphology and high levels of CD45 and major histocompatibility complex (MHC) class II after SAH. After overexpression of CX3CL1/CX3CR1, the level of CD45 and MHC class II and the release of inflammatory factors tumor necrosis factor α, interleukin 1α and complement 1q were significantly decreased. There was also increased neuronal degeneration and behavior dysfunction after SAH, both of which were inhibited by CX3CL1/CX3CR1 overexpression. Additionally, we found that the delivery of exosomal miR-124 from neurons to microglia was significantly reduced after SAH, accompanied by an increase in C/EBPα expression, and was inhibited by CX3CL1/CX3CR1 overexpression. In conclusion, the CX3CL1/CX3CR1 axis may play protective roles after SAH by promoting the delivery of exosomal miR-124 to microglia and attenuate microglial activation and neuroinflammation.

**Conclusions:**

CX3CL1/CX3CR1 axis may be a potential intervention target for the inhibition of SAH-induced EBI by promoting exosome transport of miR-124 to microglia.

## Background

Subarachnoid hemorrhage (SAH) is a relatively serious acute nervous system disease with high morbidity and mortality [1, 2]. There have been a large number of studies for seeking simpler and more efficient treatment methods, but the results are not satisfactory. Early brain injury (EBI), a critical window for determining disease progression, has also received increasing attention but has not yet been fully explored [3, 4]. Neuroinflammation is one of the main pathological processes in EBI, which is closely related to the activation of microglia [5]. Microglia are important immune cells, accounting for about 10% of the total number of cells in the central nervous system (CNS). They are the first and most important line of defense against CNS insults [6]. After SAH, a large number of microglia are activated and release inflammatory factors, which would lead to inflammatory responses and aggravate the neurological deficit [7–9].

MicroRNAs (miRNAs) are a class of small, non-coding RNA molecules and involved in the regulation of gene expression at the post-transcriptional level [10–12]. Emerging research shows that miRNAs can be secreted and delivered into recipient cells to inhibit the translation of target genes and thereby affecting the activities of cells [13]. MicroRNA-124 (miR-124) is currently the most abundant miRNA found in neurons. It has been reported that miR-124 participates in regulating microglial activation [14–16]. Exosomes, also referred to as intraluminal vesicles, are secreted by all cell types [17]. Exosomes are microvesicles with a diameter of 30–150 nm that originate from multivesicular bodies and can participate in intercellular communication by transmitting intracellular proteins, messenger RNAs, miRNAs, and long non-coding RNAs [18–20]. Recent studies have shown that exosomes play an important role in the delivery of miR-124 [16, 21, 22]. Therefore, exosomal miR-124 from neurons may play a role in microglia after SAH.

CX3CL1 (also called fractalkine) is a class of chemokine and the only member of the CX3C group [23]. CX3CL1 is a unique chemokine that binds to its only receptor, CX3CR1 [24]. In the normal CNS, CX3CL1 and its receptor CX3CR1 form signaling network between neurons and microglia [25, 26]. One study reveals that the CX3CL1/CX3CR1 axis facilitates exosome transport of miR-124 from neurons to microglia [16]. Moreover, many studies have confirmed that the CX3CL1/CX3CR1 axis plays an important role in regulating microglial activation and neuroinflammation [26, 27].

These findings suggest that targeting the CX3CL1/CX3CR1 axis may provide new insights into the inhibition of SAH-induced EBI. However, the relationship between CX3CL1/CX3CR1 axis, microglia, and miR-124 in SAH remain obscure. Therefore, one of the purposes of this study was to elucidate the effect of the CX3CL1/CX3CR1 axis on SAH. Moreover, we aim to examine the role of the CX3CL1/CX3CR1 axis in the delivery of miR-124 and to provide a new direction for the treatment of SAH.

## Materials and methods

### Patients

The study protocol was reviewed and approved by the Ethics Committee of the First Affiliated Hospital of Soochow University. Brain tissue samples were obtained from eight patients aged from 40 to 90 years who were willing to provide written informed consent. Five non-SAH brain samples were obtained from patients with a brain tumor and without any history of SAH. During neurosurgical operations for tumor treatment, normal cortical tissue that was removed to gain access to the tumor was collected. Histological examination showed no tumor infiltration in the tissue (data not shown). SAH brain tissues were obtained from three post-SAH patients. The clinical parameters and medical images of the patients are shown in Supplemental Figure 1 and Supplemental Table 1. Both brain tumor and SAH were diagnosed by neurosurgeons and radiologists based on physical examination and neuroimaging. Due to ethical considerations, the operators of subsequent studies on the excised tissue did not include neurosurgeons involved in the diagnosis and surgical treatment. The brain samples were stored in liquid nitrogen for western blot detection.

### Experimental animals

Male SD rats weighing 300–350 g were purchased from the Zhaoyan New Drug Research Center (Suzhou, China) Co., LTD. They were housed in a suitable living environment with a 12-h dark-light cycle and given adequately qualified feed and drinking water. The study was approved by the Ethics Committee of the First Affiliated Hospital of Suzhou University.

### SAH model and grade

**Fig. 1:**
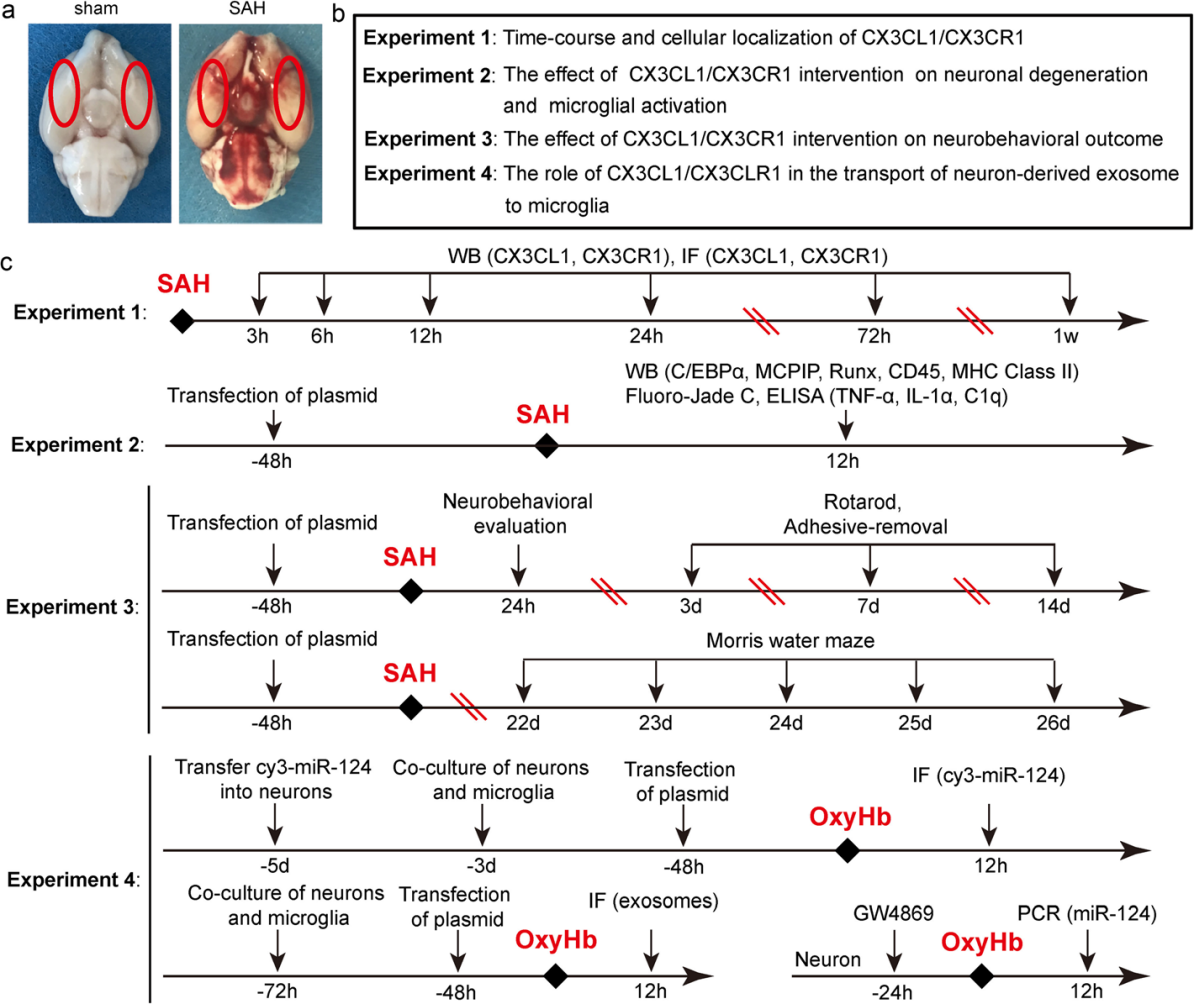
**a** Schematic representation of brain tissues of rats including sham and SAH group. **b**, **c** The detailed design of the entire animal experiment.

In this experiment, the SAH model was established by injection of autologous arterial blood into the prechiasmatic cistern. First, after being anesthetized, the rat was fixed on the stereotaxic frame (Anhui Zhenghua Biological Equipment Co., Ltd., Anhui, China). After disinfection, a median incision was made to expose the periosteum. Then, a hole was drilled 7.5 mm anterior to the bregma and 3 mm beside the midline. The needle was advanced 11–12 mm into the prechiasmatic cistern at an angle of 45 in the sagittal plane. Then, 300 μl of autologous arterial blood collected from the femoral artery was injected in 20 s. After SAH model operations, the rats were immediately injected with 5 ml physiological saline solution. The rats in the sham group were injected with 300 μl of physiological saline solution to avoid dehydration and the room temperature was kept at 23 ± 1 °C. Vital signs were monitored continuously, and the rectal temperature was kept at 37 ± 0.5 °C. As shown in Fig. 1a, the inferior basal temporal lobe was stained with blood after SAH.

The subarachnoid hemorrhage grade was evaluated using a previously established scoring method with 0–18 points scaling the degree of bleeding and was carried out by two investigators who were blind to the experiments [28]. The bases of the brain were photographed, and six segments of the basal cistern base were administered with a grade from 0 to 3 (0, no SAH; 1, minimal subarachnoid blood; 2, mediocre blood with visible arteries; 3, blood clots covering all arteries). In sham-operated rats, the score was consistently 0. Rats with SAH grading scores < 8 were excluded and replaced.

### Neuron and microglia culture

#### Primary neuron- and microglia-enriched cultures

The primary neurons were extracted from the brain of fetal rats. After the meninges and blood vessels were peeled off, the brain tissues were digested with 0.25% trypsin (with EDTA) for 5 min at 37 °C. After washing 3 times with phosphate-buffered saline (PBS), the suspension of the brain tissue was centrifuged at 1500 rpm for 5 min. The cells were resuspended in Neurobasal-A medium containing 2% B27, 2-mM l-glutamine, 50-U/ml penicillin, and 50-U/ml streptomycin (all from Gibco, USA). After counting, the cells were plated onto 6- or 12-well plates (Corning, USA) pretreated with poly-d-lysine (Sigma, USA). Plates were maintained at 37 °C under 5% CO_2_ and humidified conditions, and half of the medium was changed every 2 days.

For primary microglia cultures, the preparation of the cells was the same as the neurons. The difference was that the medium of the cells was DMEM/F12 containing 10% fetal bovine serum, 1 mM sodium pyruvate, 2 mM l-glutamine, 100 mM nonessential amino acids, 50 U/ml penicillin, and 50 mg/ml streptomycin (all from Gibco, USA). Then, cells were plated onto 150 cm^2^ culture flask pretreated with poly-d-lysine (Sigma, USA), and half the medium was changed every 2 days. Approximately 2 weeks after initial seeding, a confluent monolayer of glial cells was achieved. Microglia were separated from astrocytes by shaking the flask and collected by centrifuging.

### Co-culture of neurons and microglia

The whole process was divided into two parts. The first part: primary neuron-and microglia enriched cultures. Before extracting neurons and microglia, we first prepared 6- or 12-well plates and flasks treated with poly-d-lysine. Then, the neurons and microglia were separately cultured in 6- or 12-well plates and flasks as in the same manner described above. The second part: After the microglia in the flask were shaken, they were directly plated onto the 6- or 12-well plates of neurons after centrifuging and counting.

### Experimental design

First, we examined the expression of CX3CL1 and CX3CR1 in the brain tissues of the human. Then, the animal experiment was carried out. In order to ensure the accuracy of the experimental results, we first successfully established the SAH models (Fig. 1a). The animal experiment was divided into 4 parts (Fig. 1b, c). We used Random Number Generator (Stat Trek) to select random samples, which was available on the website: http://stattrek.com/statistics/random-number-generator.aspx. In experiment 1, 84 rats (102 rats were used, 88 rats survived after the surgery, and 4 rats were excluded) were divided into 7 groups, with 12 rats per group: sham group and six experimental groups were arranged by time 3 h, 6 h, 12 h, 24 h, 72 h, and 7 days after SAH. When the set time was reached, the rats were sacrificed and the brain tissue was collected for the time course study. In experiment 2, we exploited the time period at 12 h after SAH based on the results of experiment 1. A total of 48 rats (62 rats were used, 52 rats survived after the surgery, and 4 rats were excluded) were divided into 4 groups of 12 rats each: sham group, SAH group, and SAH + Vector group, SAH + Over-CX3CL1/CX3CR1 group. All rats were sacrificed 12 h after SAH, and the brain tissue and serum were separately collected for western-blotting, fluoro-Jade C (FJC) staining, and enzyme-linked immunosorbent assay (ELISA). Experiment 3 was aimed to test the role of CX3CL1/CX3CR1 on the behavioral ability of rats. A total of 40 rats (51 rats were used, 44 rats survived after SAH, 4 rats were excluded) were divided into 4 groups: sham group, SAH group, SAH + Vector group, and SAH + Over-CX3CL1/CX3CR1 group. The rats in each group were used for ethological testing, including neurobehavioral scores, adhesive-removal test, rotarod test, and Morris water maze test. In experiment 4, primary-cultured neurons and microglia were used to verify potential underlying mechanisms. In addition to primary neuron- and microglia-enriched cultures, we also created an environment in which neurons and microglia were co-cultured to make the two symbiotic. Here, we simulated the SAH environment by adding oxyhemoglobin (OxyHb; 10 μM) to the medium. After a series of interventions, changes in exosome-mediated transport of miR-124 between neurons and microglia were observed by immunofluorescence staining and polymerase chain reaction (PCR).

### Drug administration

Based on the former study, the GW4869 (D1692, Sigma, USA) is a commonly used drug that inhibits the production of exosomes [29]. GW4869 is dissolved in DMSO (Beyotime, China) and then diluted in culture supernatant to achieve concentration at 20 μM in a culture medium. The culture neurons were treated with GW4869 at 37 °C for 24 h, then the culture supernatants were harvested for collecting exosomes.

### Transfection of the plasmid in vivo

In this process, we used two plasmids: the plasmid of CX3CL1 and the plasmid of CX3CR1. We co-transfected them into the rat brains through Entranster—in vivo DNA transfection reagent (Engreen, China). According to the manufacturer’s instructions, 5 μl plasmid (2.5 μl of CX3CL1 plasmid and 2.5 μl CX3CR1 plasmid) or empty Vector was dissolved in 10 μl Entranster—in vivo DNA transfection reagent. After standing at room temperature for 15 min, the mixed 15 μl of the solution was injected intracerebroventricularly at 48 h before SAH.

### Transfection of the cy3-miR-124 in vitro

Transfection of the cy3-miR-124 into neurons was performed using lipofectamine 3000 Transfection Reagent (L3000-015, Invitrogen). After 48 h, the neurons were further processed.

### Reagents

Anti-CX3CL1 antibody (ab25088), anti-CX3CR1 antibody (ab8021), anti-CX3CL1 antibody (ab25088), anti-CX3CR1 antibody (ab8020), anti-CD45 antibody (ab8216), anti-MHC class II antibody (ab23990), anti-Runx1 antibody (ab23980), anti-Iba1 antibody (ab5076), anti-NSE antibody (ab53025), and anti-NeuN (ab104224) were from Abcam (USA). Anti-MCPIP antibody (sc-515275) and β-tubulin (sc-9014) were obtained from Santa Cruz Biotechnology (USA). Anti-C/EBPα antibody (2295), anti-rabbit-IgG-HRP (7074s), and anti-mouse-IgG-HRP (7076s) were obtained from the Cell Signaling Technology (USA). Alexa Fluor-488 donkey anti-rabbit IgG antibody (A21206), Alexa Fluor-555 donkey anti-mouse IgG antibody (A31570), Alexa Fluor-555 donkey anti-goat IgG antibody (A21432), and Alexa Fluor-633 donkey anti-goat IgG antibody (A21082) were from the Life Technologies. RBFOX3/NeuN antibody [Alexa Fluor-405] (NBP1-77686AF405) was from Novus (USA).

### Isolation and collection of exosomes

This detection was performed according to the manufacturer’s instructions (EXOQ20A-1, System Biosciences, USA). Supernatants from cultured neurons were collected and centrifuged at 3000*g* for 15 min to remove any cells and cell debris, and then supernatants were transferred to a fresh tube. Then, according to the instructions, the exosome isolation reagent was added to the supernatants and allowed to stand at 4 °C overnight (at least 12 h). Finally, the mixture was centrifuged at 1500*g* for 30 min.

### Determination of exosomal miRNA abundance

Exosomal miR-124 abundance was determined by real-time quantitative PCR (RT-qPCR). First, the total RNA was extracted from the precipitated exosomes obtained above by TRIzol. According to the manufacturer’s instructions (ZK00805, ShineGene Molecular Biotech, China), the RNA was reverse transcribed to complementary DNA (cDNA), and RT-PCR was performed. GAPDH served as loading controls. Primers used in RT-qPCR were obtained from the ShineGene Molecular Biotech (China). The qPCR amplification reaction was performed with a volume of 50 μl, containing 25 μl 2 × Hotstart Fluo-PCR mix, 1 μl each primer (25 pmol/l), 0.5 μl probe (25 pmol/l), 8 μl DEPC water, and 1 μl cDNA. The PCR amplification was as follows: denaturation at 94 °C for 4 min, followed by 40 cycles of 94 °C for 20 s and 60 °C for 30 s with continuous fluorescence measurement. Quantification was performed by using a comparative CT method (2^−ΔΔCT^). All samples were analyzed in triplicate [30, 31].

1. MiR-124 sequences: Forward primer: 5′ TGTAAGGCACGCGGTG 3′

Reversed primer: 3′ GTGCAGGGTCCGAGGT 5′

(2) GAPDH sequences: Forward primer: 5′ TGGAGTCTACTGGCGTCTT 3′ Reversed primer: 3′ TGTCATATTTCTCGTGGTTCA 5′

### Western-blotting analysis

After lavaging of the brain tissue with PBS, the temporal base brain tissues were taken out. After lysis and standing, the brain tissue was centrifuged (12000 rpm, 5 min, 4 °C). The supernatant protein concentration was measured using the 96-well Cell Culture Cluster and enhanced BCA Protein Assay Kit (P0010S, Beyotime, China). After adding a loading buffer to the trimmed protein sample, it was heated at 100 °C for 5 min. Then, the protein samples were loaded on SDS-polyacrylamide gels and were then separated and electrophoretically transferred to polyvinylidene-difluoride membranes (IPVH00010, Millipore Corporation, USA). After blocking with 5% non-fat milk at room temperature for 1 h, the membranes were incubated overnight at 4 °C with primary antibodies. β-tubulin was used as a loading control. After washing 3 times with PBST (PBS + 0.1%Tween-20), the membranes were incubated at room temperature for 1.5 h with secondary antibodies against mouse or rabbit. Finally, the protein bands were visualized using an Enhanced Chemiluminescence (ECL) Kit (Clinx, China) and digitalized with a ChemiScope 5300 Chemiluminescence imaging system (Clinx). Blots were imaged and quantified using the ImageJ software (NIH, Bethesda, MD, USA) [32, 33].

### Immunofluorescent analysis

After lavaging with PBS, the total coronal sections containing the temporal base brain tissue were fixed with 4% paraformaldehyde, embedded in paraffin, and sectioned. After heating and dewaxing, the sections were incubated overnight at 4 °C with primary antibodies. For cells, they could be incubated as long as they were fixed by 4% paraformaldehyde. After washing 3 times with PBST, the sections were incubated with secondary antibodies at 37 °C for 1 h. After washing three times with PBST, the sections were sealed with 4′,6-diamidino-2-phenylindole (DAPI) Fluoromount-G@ (Southern Biotech, USA). Finally, sections were observed by fluorescent microscope (OLYMPUS BX50/BX-FLA/DP70; Olympus Co., Japan), laser scanning confocal microscope (ZEISS LSM 880, Carl Zeiss AG, Germany) and stimulated emission depletion microscopy (Jiangsu Key Laboratory of Medical Optics, Suzhou Institute of Biomedical Engineering and Technology, China) [34].

### FJC staining

The first step was the same as that for immunofluorescent analysis. After heating and dewaxing, the sections were incubated in 80% alcohol with 20% sodium hydroxide for 5 min, 70% alcohol for 2 min, distilled water for 2 min, 0.06% K permanganate for 10 min and 0.0004% FJC-working solution for 20 min, and were then dried in an incubator (50–60 °C) for 15–30 min. After drying, the sections were incubated in xylene for 2 min. Then, they were sealed with neutral gum. Finally, sections were observed by a fluorescent microscope (OLYMPUS BX50/BX-FLA/DP70; Olympus Co., Japan).

### ELISA

According to the manufacturer’s instructions, the levels of tumor necrosis factor α (TNF-α), interleukin 1α (IL-1α), and Complement 1q (C1q) in the serum were measured using a specific ELISA Kit (Bio-Swamp, China).

### Neurobehavioral scores

At 24 h after SAH, the rats were examined for behavioral impairment using an established scoring system. This scoring system consisted of three parts: appetite, activity, and neurologic defects [35].

### Adhesive-removal test

This test was used to measure motor coordination and sensory neglect after SAH. First, we placed the rat in a glass box for a period of time and attached a circular sticker to the palm of each forepaw. The time at which the rat removed all the stickers was recorded. All rats were trained daily for 3 days prior to modeling. Then, the test was carried out 1 day before modeling and on the 1st, 3rd, 7th, and 14th days after the SAH.

### Rotarod test

This test was to assess the locomotor ability of the rats by a rota-rod cylinder (ZH-300B, Anhui Zhenghua Biological Equipment Co., Ltd, China). The rat was placed on the horizontal axis that had been set at a constant rate from 4 to 30 rpm within 1 min. When the rat dropped or gripped the device around for two revolutions, the test was finished and the time animals remained on the rotarod was recorded. As with adhesive-removal test, all rats were trained 3 days prior to modeling. The test was also carried out 1 day before modeling and on the 1st, 3rd, 7th, and 14th days after the SAH.

### Morris water maze

The method of the Morris water maze test has been previously described [36]. The test was carried out in a circular tank of 180-cm diameter and 50-cm depth. A circular platform with the diameter of 12 cm was placed 2 cm below the surface of the water. All rats were trained for 4 days from the 18th day after modeling, 3 times a day. Each training lasted 1 min and was separated by 5 min every two times. If the rat could board the platform within 1 min, it would be allowed to stay for 15 s; on the contrary, it would be guided to board the platform. The testing phase took place from the 22nd day to the 26th day after SAH.

### Statistical analysis

GraphPad Prism 7.0 software (GraphPad, USA) was used for statistical analysis. Neurobehavioral scoring is shown as the median with the interquartile range, and the Mann-Whitney *U* test was used to compare scores among groups. All other data are reported as the mean ± SD. One-way or two-way ANOVA was used for multiple comparisons, and Bonferroni’s or Tukey’s post hoc test was used for comparison between two pairs in multiple groups. *P* < 0.05 indicated a statistically significant difference. Specific statistics are shown in Supplemental Table 2.

## Results

### General observation

Throughout the study, the mortality rate of rats in the sham group was 0% (0/34 rats) and was 17.1% (31/181 rats) in the SAH groups (Supplemental Table 3).

### CX3CL1 and CX3CR1 protein levels decreased in the brain tissues of patients after SAH

**Fig. 2:**
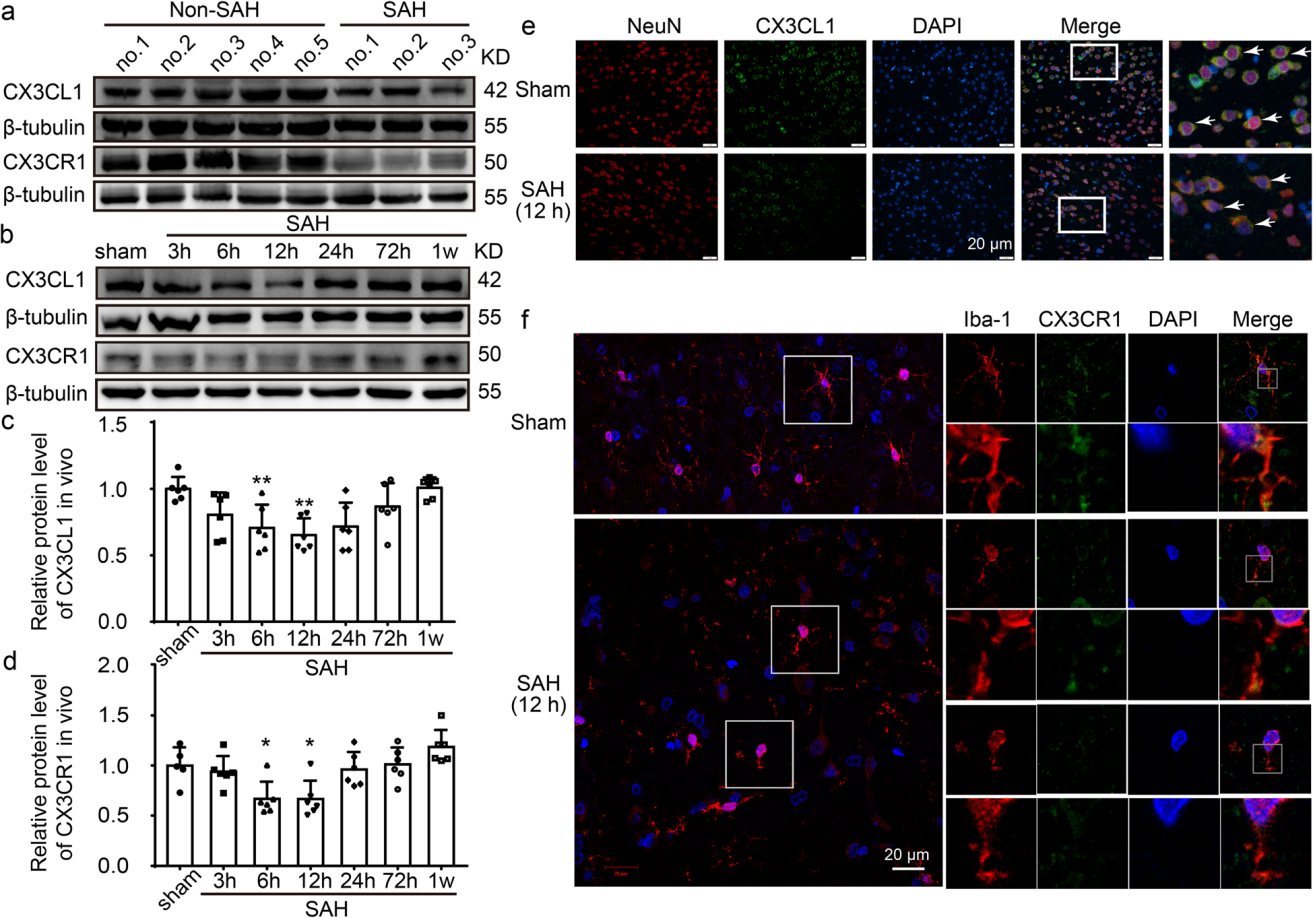
The protein levels of CX3CL1 and CX3CR1 in the brain tissues of patients and rats after SAH. **a** Western-blot analysis of protein levels of CX3CL1 and CX3CR1 in brain tissues of patients. **b** Western-blot analysis showed the protein levels of CX3CL1 and CX3CR1 at 3 h, 6 h, 12 h, 24 h, 72 h and 7 days after SAH. **c**, **d** Quantification of protein levels of CX3CL1 and CX3CR1 at different time points. **e** Double-immunofluorescence analysis was performed with CX3CL1 (green) and a neuronal marker (NeuN, red) in sections. Nuclei were fluorescently labeled with 4′-6-diamidino-2-phenylindole (DAPI) (blue). Arrows point to CX3CL1-positive neurons. Scale bar = 20 μm. **f** Double-immunofluorescence analysis was performed with CX3CR1 (green) and a microglia marker (Iba-1, red) in sections. Nuclei were fluorescently labeled with DAPI (blue). Scale bar = 20 μm. In (**c**, **d**), the mean values for the Sham group were normalized to 1.0, and data are mean ± standard deviations (SD), *n* = 6. *P < 0.05, **P < 0.01 vs sham group

To detect the protein levels of CX3CL1 and CX3CR1 after SAH, western blot analysis of protein samples from the brain tissues of patients was performed (Fig. 2a). The results showed that both CX3CL1 and CX3CR1 were expressed in the brain tissues of non-SAH and SAH patients. Compared with non-SAH group, the protein levels of CX3CL1 and CX3CR1 were reduced in SAH patients.

### CX3CL1 and CX3CR1 protein levels decreased in the brain tissues of rats after SAH

After observing the changes in proteins CX3CL1 and CX3CR1 in the human brain, we further examined their levels in the rat brain. Western blot analysis showed that, compared with the sham group, the protein level of CX3CL1 was significantly decreased at 12 h after SAH (*P* < 0.01, Fig. 2b, c). Similarly, the protein level of CX3CR1 also decreased and reached the lowest level at 12 h (*P* < 0.05, Fig. 2b, d). Previous study has demonstrated that, in the CNS, CX3CL1 was expressed predominantly in neurons, and its receptor CX3CR1 was expressed solely on microglia [37]. Double immunofluorescence was performed to distinguish the changes in cell type-specificity expression of CX3CL1 and CX3CR1 after SAH. Compared with the sham group, the protein levels of CX3CL1 in neurons and CX3CR1 in microglia showed significant decreases at 12 h after SAH (Fig. 2e, f). In addition to the expression of target proteins, we also observed a significant change in the morphology of microglia. After SAH, microglia lost processes and increased in size (Fig. 2f).

### CX3CR1/CX3CL1 overexpression inhibited SAH-induced neuronal degeneration

**Fig. 3:**
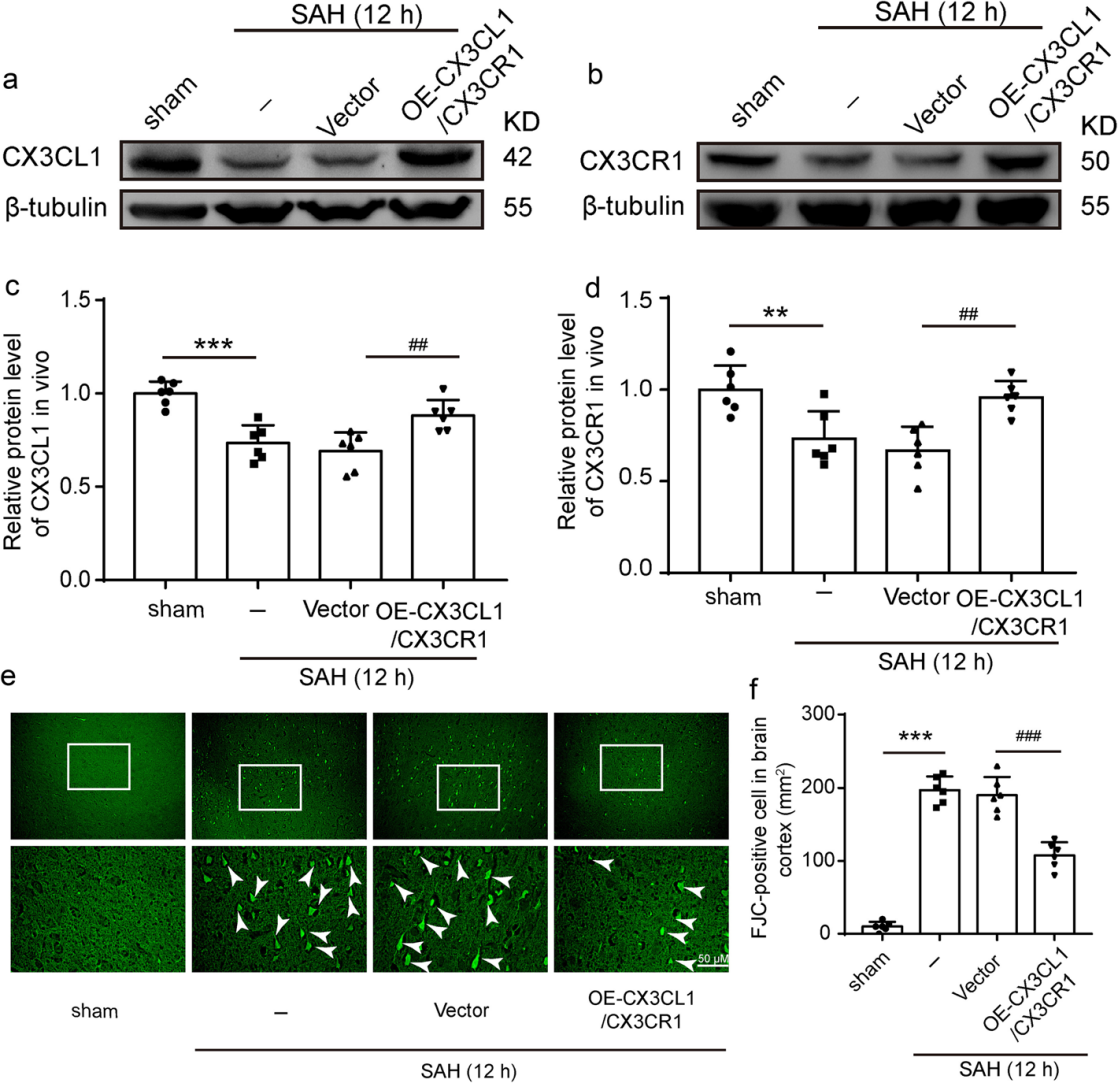
The effect of CX3CL1/ CX3CR1 overexpression on brain injury after SAH. **a, b** Western-blot analysis showed the protein levels of CX3CL1 and CX3CR1 under plasmid of CX3CL1/CX3CR1 intervention. **c**, **d** Quantification of CX3CL1 and CX3CR1 protein levels. **e** Fluoro-Jade C (FJC) staining (green) was performed to assess neuronal degeneration at 12 h after SAH and arrows pointed to FJC-positive cells. **f** Quantitative analysis of FJC-positive cells/mm^2^ in brain sections in each group. Scale bar = 50 μm. In **c**, **d**, the mean values for the Sham group were normalized to 1.0. In (**c**, **d**, **f**), all data are mean ± SD, *n* = 6. **P < 0.01, ***P < 0.001vs sham group; ^##^*P* < 0.01, ^###^*P* < 0.001vs SAH + vector group

To investigate the role of CX3CL1 and CX3CR1 on EBI induced by SAH, we used FJC staining to detect the effect of CX3CL1 and CX3CR1 overexpression on neuronal degeneration in the brain at 12 h after SAH. We simultaneously upregulated CX3CL1 and CX3CR1 in the brain tissues by plasmid transfection, and the transfection efficiency of plasmid was verified by western blotting analysis (Fig. 3a, b). CX3CL1 and CX3CR1 protein levels reduced at 12 h after SAH compared with the sham group (*P* < 0.001 and *P* < 0.01, Fig. 3c, d), and they were significantly upregulated by overexpression of CX3CL1 and CX3CR1 (*P* < 0.01, Fig. 3c, d). Compared with the sham group, the number of FJC-positive cells significantly increased in the SAH group (*P* < 0.001, Fig. 3e, f), which was significantly decreased after CX3CR1/CX3CL1 overexpression (*P* < 0.001, Fig. 3e, f).

### CX3CR1/CX3CL1 overexpression improved the recovery of neurological function deficits after SAH

**Fig. 4:**
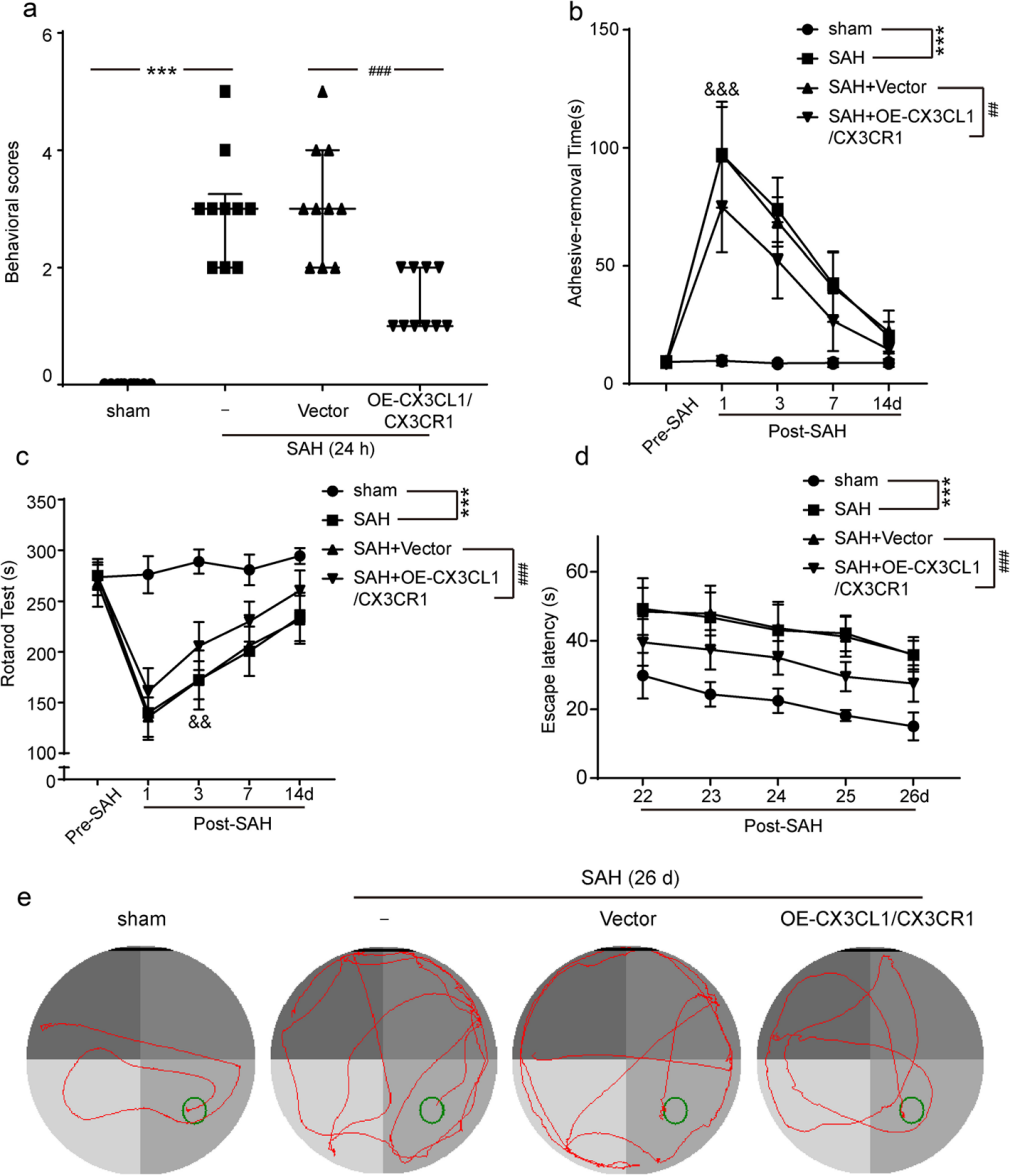
The effect of CX3CL1/ CX3CR1 overexpression on recovery of neurological function of rats after SAH. **a** Neurobehavioral scores, *n* = 10. **b** Adhesive-removal test, *n* = 10. **c** Rotarod test, *n* = 10. **d** Time to reach the submerged platform in the water maze from 22 to 26 days after SAH, *n* = 6. **e** The typical swim path of rats in the Morris water maze test at 26 days after SAH. In **a**, the data are median and interquartile range; in **b**, **c**, and **d**, all data are mean ± SD. ***P < 0.001 vs sham group; ^##^*P* < 0.01, ^###^*P* < 0.001 vs SAH + vector group

To examine whether CX3CR1/CX3CL1 overexpression benefits neurological behavior, the behavioral activity of all rats was examined by behavioral scoring at 24 h after SAH. As shown in Fig. 4a, compared with the sham group, rats showed severe neurological impairment after SAH (*P* < 0.001), which was significantly alleviated by CX3CR1/CX3CL1 overexpression (*P* < 0.001).

We also tested the effect of CX3CL1/CX3CR1 overexpression on the sensory function of rats. At 1, 3, 7, and 14 days post-SAH, all rats were subjected to the adhesive-removal test, in which fine sensorimotor function and forelimb coordination were assessed. Post-operative rats took longer to remove the stickers than the sham group (*P* < 0.001, Fig. 4b). But when CX3CR1/CX3CL1 was upregulated, this duration was significantly shortened (*P* < 0.01, Fig. 4b). In addition to the above, we also evaluated the locomotor function of the rat by the rotarod test. As shown in Fig. 4c, compared with the sham group, the locomotion of the rats after SAH was significantly damaged, but the overexpression of CX3CL1/CX3CR1 promoted the recovery of function (both *P* < 0.001). Moreover, we also found that the recovery of sensory function took precedence over the locomotor function.

To further examine the long-term effect of CX3CL1/CX3CR1 overexpression on SAH outcomes, we performed the Morris water maze test on rats during days 22–26 after SAH onset. Compared with the sham group, the learning and memory functions of rats after SAH were severely impaired. However, rats spent less time finding the ultimate goals in CX3CL1/CX3CR1 overexpression group (both *P* < 0.001, Fig. 4d). The representative trajectory in different groups is shown in Fig. 4e.

### CX3CL1/CX3CR1 overexpression mediated SAH-induced activation of microglia and inflammatory response

**Fig. 5:**
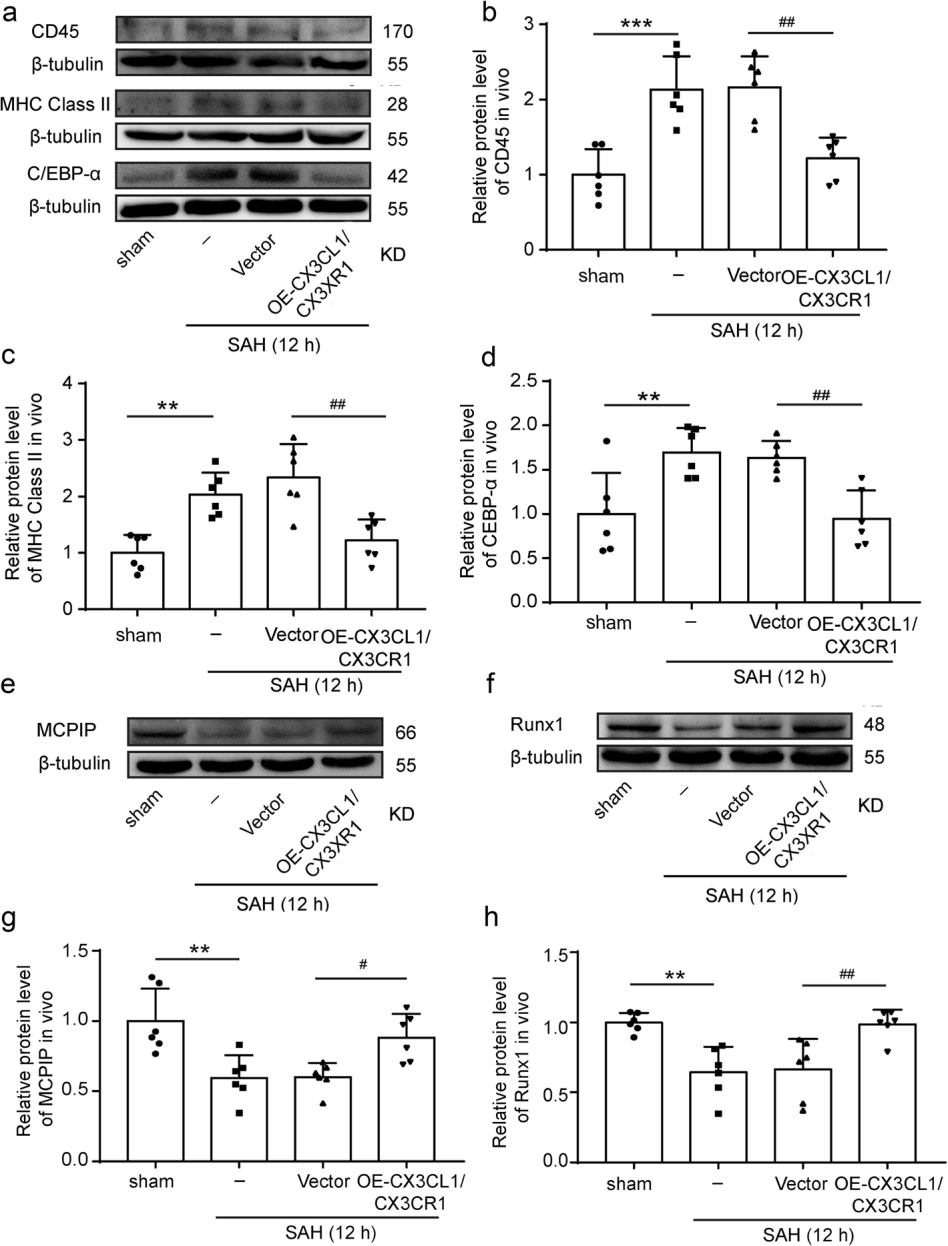
The effect of CX3CL1/ CX3CR1 overexpression on the activation of microglia. **a** Western-blot analysis and quantification of the protein levels of CD45, MHC class II and CCAAT-enhancer-binding protein α (C/EBP-α). **b–d** Quantification of CD45, (MHC) class II and C/EBP-α. **e**, **f** Western-blot analysis of monocyte chemotactic protein-induced protein 1 (MCPIP1) and RUNX1 in the brain tissues of rats after SAH. In **b**, **c**, **d**, **g**, and **h**, mean values for the sham group were normalized to 1.0, and data are mean ± SD, *n* = 6. **P < 0.01, ***P < 0.001 vs sham group; ^#^*P* < 0.05, ^##^*P* < 0.01vs SAH + vector group

**Fig. 6:**
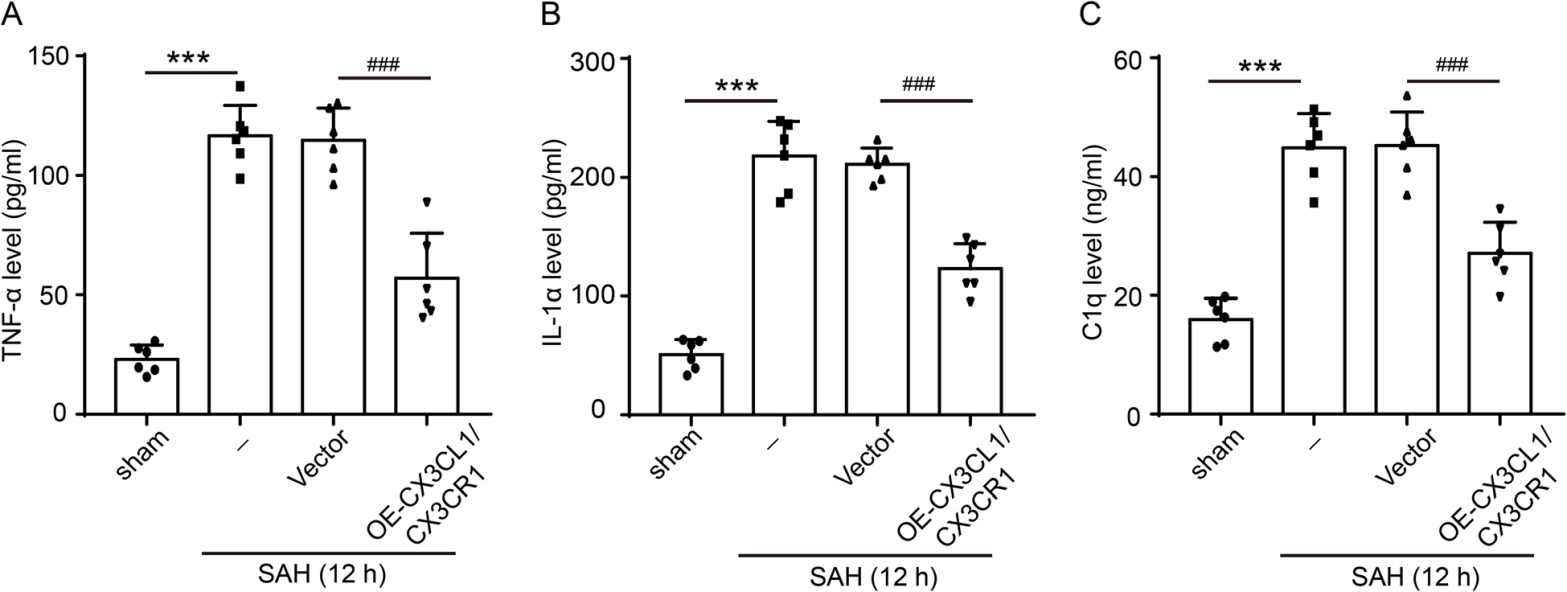
The effect of CX3CL1/CX3CR1 overexpression on SAH-induced inflammation. Concentrations of pro-inflammatory cytokines of **a** tumor necrosis factor α (TNF-α), **b** interleukin 1α (IL-1α), and **c** Complement 1q (C1q) in serum were assayed after SAH. Data are mean ± SD, *n* = 6. ***P < 0.001 vs sham group; ^###^*P* < 0.01 vs SAH + vector group

To further ascertain the effects of CX3CL1/CX3CR1 overexpression on activation of microglia, we observed trends in CD45, leukocyte common antigen (LCA), and major histocompatibility complex (MHC) class II, which can be upregulated after microglia activated [38–40]. After SAH, the levels of CD45 and MHC class II in the brain tissues were significantly increased (*P* < 0.001 and *P* < 0.01, Fig. 5a–c). When we artificially upregulated CX3CL1 and CX3CR1 in the rat brain tissues after SAH, the expression of CD45 and MHC class II was significantly inhibited (*P* < 0.01, Fig. 5a–c). In addition to the above two markers, the protein levels of CCAAT-enhancer-binding protein α (C/EBP-α), monocyte chemotactic protein-induced protein 1 (MCPIP1), and Runx1 in brain tissues were also detected by western blot analysis. C/EBP-α was widely expressed and upregulated upon microglial activation [16], while MCPIP and Runx1 were negative regulators of macrophage activation [41, 42]. As shown in Fig. 5a, d, CX3CL1/CX3CR1 overexpression could suppress the SAH-induced C/EBP-α expression (*P* < 0.01). In contrast to C/EBP-α, the expression of MCPIP and Runx1 was inhibited after SAH (*P* < 0.01, Fig. 5e–h), and the overexpression of CX3CL1/CX3CR1 could reduce this inhibition (*P* < 0.05 and *P* < 0.01, Fig. 5e–h). Finally, inflammatory cytokines, including TNF-α, IL-1α, and C1q, were found to be significantly higher in the serum of the SAH group than in that of the sham group (*P* < 0.001, Fig. 6a–c), which was significantly reduced by CX3CL1/CX3CR1 overexpression (*P* < 0.001, Fig. 6a–c).

### CX3CL1/CX3CR1 overexpression promoted the delivery of exosomal miR-124 from neurons to microglia after SAH

**Fig. 7:**
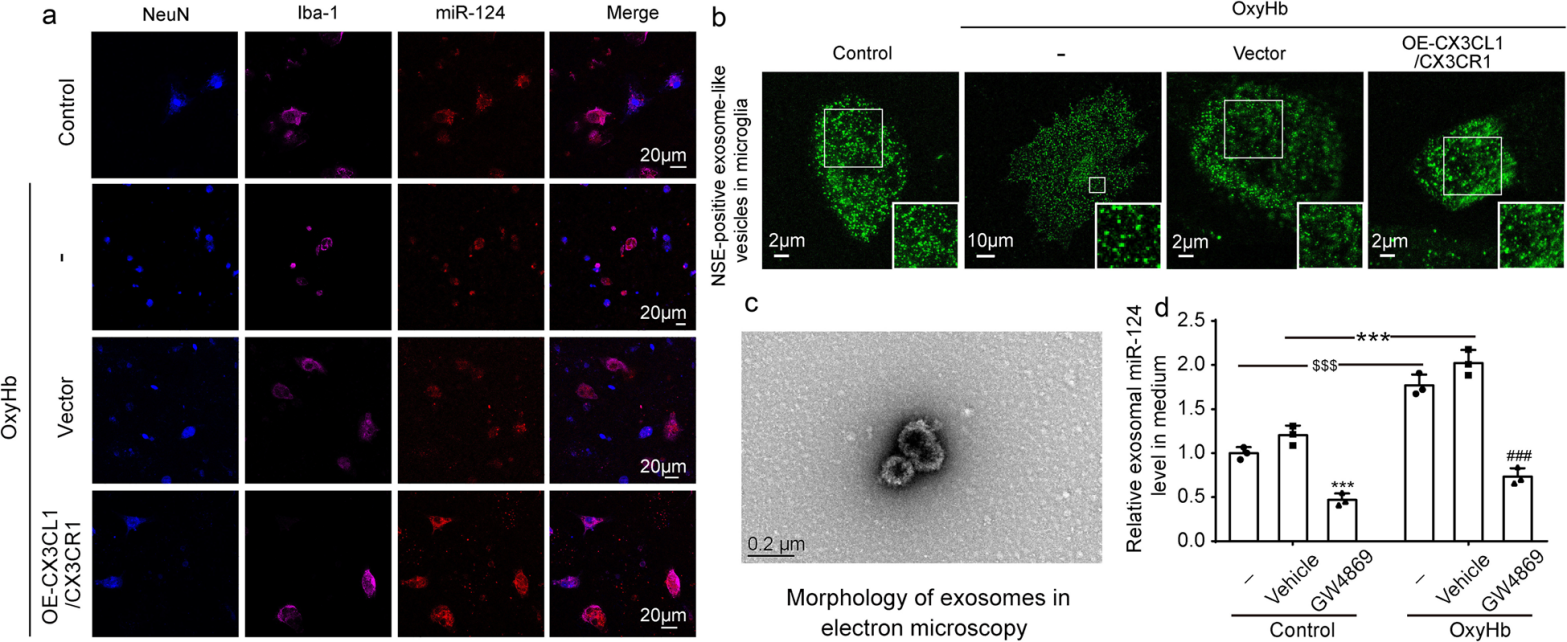
The effect of CX3CL1/CX3CR1 overexpression on the delivery of exosomal microRNA-124 (miR-124). **a** Triple-immunofluorescence analysis was performed with cy3-miR-124 (red), a neuronal marker (NeuN, blue), and a microglia marker (Iba-1, purple) in co-cultured neurons and microglia. Scale bar = 20 μm. **b** Immunofluorescence analysis was performed with neuron-specific enolase (NSE) antibodies (green) in co-cultured neurons and microglia observed by stimulated emission depletion microscopy. **c** Scanning electron micrograph of exosomes isolated from medium. **d** Real-time quantitative polymerase chain reaction (RT-qPCR) assay of the exosomal miR-124 levels in medium. In **d**, the mean values for the control group were normalized to 1.0, and all data are mean ± SD, *n* = 3. ***P < 0.001 vs control + vehicle group; ^###^*P* < 0.001vs OxyHb + vehicle group; ^$$$^*P* < 0.001 vs control group

To verify the delivery of miR-124 between neuronal-microglia, we transferred the exogenous cy3-miR-124 into the neurons before the co-culture of neurons and microglia. Immunofluorescence staining showed that miR-124 had a transfer from neurons to microglia in a normal condition. When OxyHb was administered, the delivery was reduced (Fig. 7a). In addition to miR-124, we also focused on the transfer of exosome. Neuron-specific enolase (NSE) was a specific marker of neurons. Consistent with neuron-derived miR-124, there were fewer NSE-positive exosome-like vesicles in the area of microglia (Fig. 7b). After the overexpression of CX3CL1/CX3CR1, the delivery of both miR-124 and NSE-positive exosome-like vesicles was significantly increased (Fig. 7a, b).

To further determine whether miR-124 was directly delivered from neurons to microglia via exosomes, a specific chemical inhibitor of exosome, GW4869, was applied. We used GW4869 to intervene in primary neurons and examined the abundance of miR-124 in the exosomes isolated from the culture medium. First, we observed the morphology of exosomes isolated from the culture medium by electron microscopy (Fig. 7c). The RT-qPCR results suggested that the exosomal miR-124 markedly increased via OxyHb administration both in control and vehicle group (both *P* < 0.001, Fig. 7d), but the treatments with GW4869 could significantly reduce the exosomal miR-124 derived from neurons (both *P* < 0.001, Fig. 7d).

## Discussion

SAH is an extremely serious stroke disease, and SAH-induced EBI is considered the main factor leading to poor clinical outcomes. Even if they survive, most people face lifelong disabilities, and the quality of life is difficult to guarantee. In this study, we have provided new insights into the role of the CX3CL1/CX3CR1 axis in the transfer of exosomal miR-124 and in the function of microglial activation after SAH.

In SAH-induced EBI, microglia play a key role. In normal CNS, most microglia are highly active in a “surveillance and rapid response” instead of “resting” state to monitor and control the activity of neurons [43]. They exhibit a non-activated phenotype with long processes and branches, which is determined by low expression levels of CD45 and MHC class II [44, 45]. In many lesions of the CNS, microglia are activated and changed from a “watching” role to a “fighting” role with the upregulation of activation markers such as CD45 and MHC class II. Simultaneously, they change morphology from ramified to macrophage-like morphology [41]. Therefore, the phenotype of microglia is very important, which can be an indicator of their function. In this study, we observed macrophage-like changes in microglia morphology and high CD45 and MHC class II expression in the brains after SAH, demonstrating that microglia are activated. Then, activated microglia release a large number of inflammatory factors like TNF-α, IL-1α, and C1q, which in turn lead to neuroinflammation and neuronal death [46, 47].

Evidence from previous studies has shown that the expression of miR-124 correlated inversely with the activation state of microglia [14, 15, 48], where the highest level of miR-124 expression was observed in CD45^low^ MHC class II^low^ non-activated microglia [49, 50]. In this study, we found that miR-124 could be delivered from neurons to microglia in normal conditions. MiR-124 functionally regulated the activation state of microglia by targeting C/EBPα [50, 51]. C/EBPα is a key regulator of microglia quiescence. In CNS, C/EBPα was not found to be expressed in neurons, astrocytes, or resting microglia in rat brain, but was detected in activated microglia [52]. In the absence of C/EBPα, the expressions of CD11b, MHC class II, and CD86 were decreased [50]. Herein, we further demonstrated that the neuron-derived miR-124 was delivered via exosomes. Therefore, exosomal miR-124 from neurons could bind C/EBPα to regulate the phenotype of microglia after SAH.

As previously reported, cell-cell contacts in the CX3CL1/CX3CR1 axis were involved in the transfer of miR-124 [16], and this conjugation kept microglia “quiescent” [53, 54]. In CNS, CX3CL1 mainly expressed in neurons and had two different forms: one is a membrane-bound glycoprotein providing direct interaction with CX3CR1, which expressed in microglia; the other is a soluble form working as an extracellular mediator [25]. First, we found that the protein levels of CX3CL1 and CX3CR1 were significantly reduced in SAH patients’ brain. We further tested their expressions in the rat model. Consistently, the protein levels of CX3CL1 and CX3CR1 were also significantly reduced at 12 h after SAH. These results suggested that this conjugation was reduced after SAH. As shown in Figs. 5 and 7, C/EBPα, CD45, and MHC class II levels were upregulated after SAH, which were correlated with the reduction of exosomal miR-124 in microglia. When we restored the junction of CX3CL1/CX3CR1 by plasmid transfection after SAH, exosomal miR-124 could be normally delivered from neurons to microglia to target C/EBPα, thereby inhibiting the activation of microglia. These results indicated that the CX3CL1/CX3CR1 axis was involved in the signaling pathways of exosomal miR-124-C/EBPα to promote microglia quiescence.

In addition, we found that the upregulation of CX3CL1/CX3CR1 could reduce TNF-α, IL-1α, and C1q secretion under SAH conditions. Moreover, we also observed that the overexpression of CX3CL1/CX3CR1 reduced neuronal degeneration and improved short- and long-term neurological functions after SAH. These results suggested that the CX3CL1/CX3CR1 axis could play anti-inflammatory and neuroprotective roles in SAH models. These results were consistent with several previous observations. Cipriani et al. [55] found that CX3CL1 could reduce infarct volume and improve behavioral outcomes for middle cerebral artery occlusion rats. Additionally, the lacking of CX3CR1 was cytotoxic in models of amyotrophic lateral sclerosis, Parkinson’s disease, and Alzheimer’s disease [56, 57]. Therefore, CX3CL1/CX3CR1 may be a potential intervention target for SAH patients; thus, promoting the delivery of miR-124 to microglia may be a novel approach for ameliorating SAH-induced EBI.

As shown in Fig. 7d, more neuron-derived miR-124 was released via exosomes after SAH. However, the loss of the CX3CL1/CX3CR1 junction caused exosomal miR-124 not to be delivered to microglia. So, where did it go? Recently, studies have emerged that axo-myelinic synapse (AMS) composed of neurons and oligodendrocytes could transport lactic acid to axons via monocarboxylate transporters (MCTs). In addition, the axonal injury but not oligodendrocyte death after oligodendrocyte-specific removal of MCT1 suggested that this transport could be vital for axon survival [58]. MiR-124 was confirmed to selectively target MCT1 3′ UTR [59], so more exosomal miR-124 may be transported into oligodendrocyte to target MCT1 after SAH. It has been reported that in addition to CX3CL1/CX3CR1, cell-cell contact CD200/CD200R was also thought to be involved in the activation of microglia and the delivery of exosomal miR-124 [16, 60]. We also tested the levels of CD200 and CD200R in SAH models. However, compared with CD200/CD200R (date was not shown), the change of CX3CL1/CX3CR1 was more significant. Therefore, we mainly studied the role of CX3CL1/CX3CR1 in the activation of microglia and the delivery of exosomal miR-124.

This study also had some limitations. First, our experimental samples were limited, and in the future research, we will further expand the clinical sample size, especially to clarify the relationship between the expression level of miR-124 in cerebrospinal fluid and the prognosis of patients, and analyze the potential of miRNA-124 level in cerebrospinal fluid as a clinical diagnostic marker. Moreover, the animal sample size will be amplified to explore the correlation between protein levels and neurobehavioral data. Second, we only used OxyHb to simulate SAH in vitro experiment. However, there are many other relevant components in the blood, and the effects of these have not been confirmed. Third, in this experiment, we only observed the relationship between CX3CL1/CX3CR1 axis and miR-124. In addition to C/EBPα, we also observed changes in Runx and MCPIP1, the target proteins of microRNA-9 (miR-9) [42, 61], so the CX3CL1/CX3CR1 axis may affect miR-9 in microglia. Considering the sophisticated functions of CX3CL1/CX3CR1 axis, we should take more efforts to elucidate the precise mechanism underlying the CX3CL1/CX3CR1 axis in future studies. Furthermore, the glial network is an interconnected and interactive neural network, and our research only focuses on the effect of miR-124 on microglia after SAH, while the specific role of miR-124 in astrocytes and oligodendrocytes has not yet been involved.

## Conclusions

**Fig. 8:**
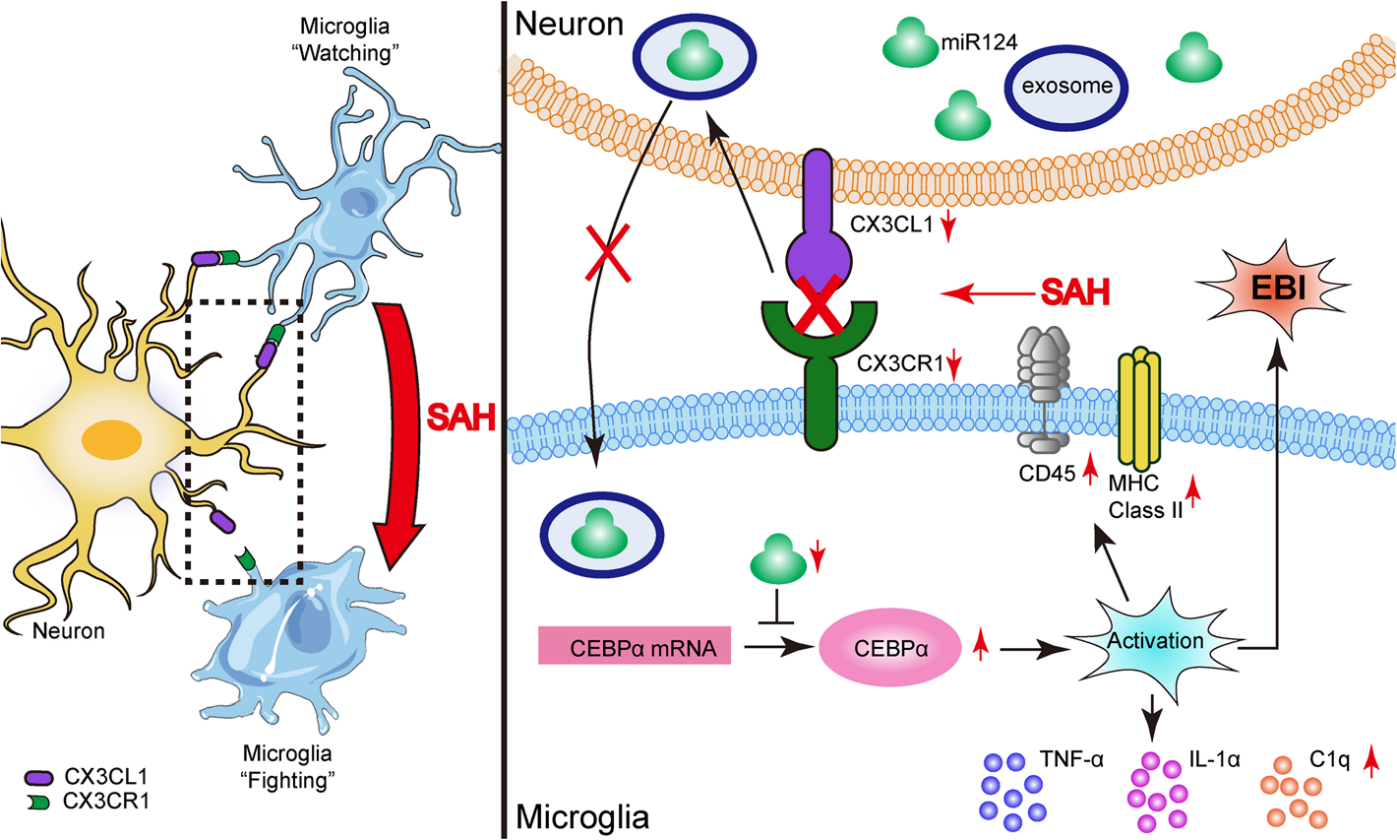
Roles of the CX3CL1/CX3CR1 axis in SAH-induced early brain injury. MicroRNA-124 (miR-124) plays a vital role in microglial activation by targeting protein CCAAT-enhancer-binding protein α (C/EBPα). CX3CL1/CX3CR1 axis is involved in the delivery of miR-124 from neurons to microglia. The protein levels of CX3CL1/CX3CR1 were significantly reduced after SAH, accompanied by an increase in C/EBPα expression. CX3CL1/CX3CR1 axis may play a protective role after SAH by promoting the delivery of exosomal miR-124 from neurons to microglia to attenuate microglial activation and neuroinflammation

This study demonstrated the role of the CX3CL1/CX3CR1 axis in inducing neuroprotection against SAH-induced EBI. This axis was closely related to exosomes to transport miR-124 from neurons to microglia. By promoting the transport of miR-124 to microglia, it regulated the expression of target protein C/EBPα of miR-124 in microglia, thereby inhibiting the activation of microglia and reducing the inflammatory response. It suggested the CX3CL1/CX3CR1 axis might be good target for improving EBI after SAH (Fig. 8).

## Supplementary information

**Additional file 1: Figure S1.** The medical images of clinical samples. **Table S1.** The information of clinical samples. **Table S2.** Statistical table. **Table S3.** Modeling situation.

## Data Availability

Data generated and analyzed as part of this study are included in the manuscript or are available upon request from the corresponding author.

## Abbreviations

AMS: Axo-myelinic synapse
CNS: Central nervous system
C/EBPα: CCAAT-enhancer-binding protein α
C1q: Complement 1q
DEPC water: Diethylpyrocarbonate water
EBI: Early brain injury
ELISA: Enzyme-linked immunosorbent assay
FJC: Fluoro-Jade C
IL-1α: Interleukin 1α
MCPIP1: Monocyte chemotactic protein-induced protein 1
MCTs: Monocarboxylate transporters
MHC: Major histocompatibility complex
MiRNAs: MicroRNAs
MiR-9: MicroRNA-9
MiR-124: MicroRNA-124
NSE: Neuron-specific enolase
OxyHb: Oxyhemoglobin
PBS: Phosphate-buffered saline
RT-qPCR: Real-time quantitative polymerase chain reaction
SAH: Subarachnoid hemorrhage
TNF-α: Tumor necrosis factor α
