# Supplementary material for: CX3CL1/CX3CR1 axis attenuates early brain injury via promoting the delivery of exosomal microRNA-124 from neuron to microglia after subarachnoid hemorrhage
Source: J Neuroinflammation. 2020 Jul 14;17:209. doi: 10.1186/s12974-020-01882-6 (PMC7362528; doi:10.1186/s12974-020-01882-6)
Supplement: Supplementary file 1 — Additional file 1: Figure S1. The medical images of clinical samples. Table S1. The information of clinical samples. Table S2. Statistical table. Table S3. Modeling situation. [file 12974_2020_1882_MOESM1_ESM.pdf]

Supplementary material

CX3CL1/CX3CR1 axis attenuates early brain injury via promoting the delivery of exosomal microRNA-124 from neuron to microglia after subarachnoid hemorrhage

Supplemental Figure 1. The medical images of clinical samples.

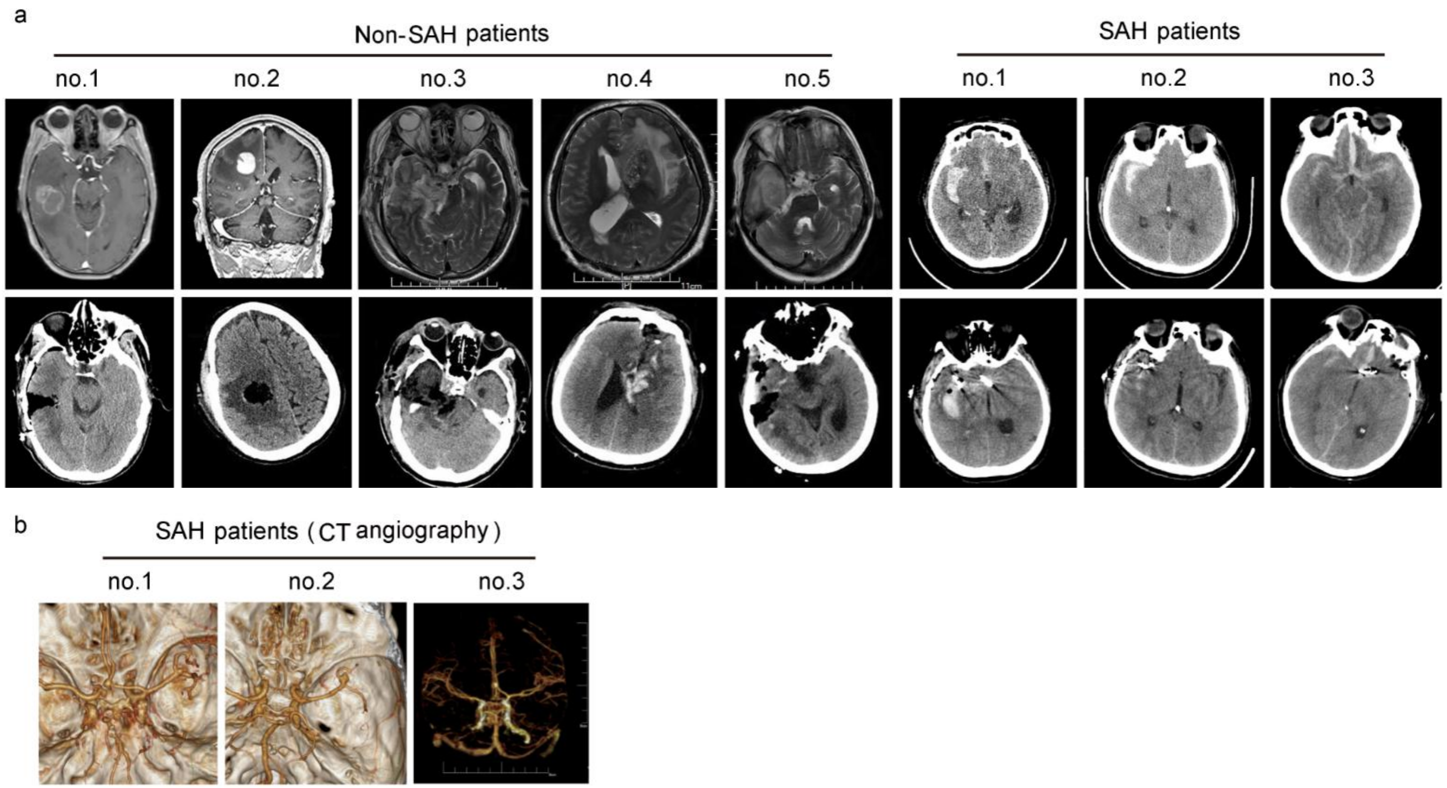

Supplemental Table 1. The information of clinical samples.

Group	No.	Age	Gender	Diagnosis	GCS score	Hunt-hess rating	Operation time (day after SAH)	Part of the sample	Prognosis
Non-SAH	1	63	Male	Metastatic encephaloma					
	2	68	Male	Metastatic encephaloma					
	3	72	Male	Meningioma					
	4	65	Female	Glioma					
	5	42	Male	Glioma					
SAH	1	72	Female	Aneurysms rupture with subarachnoid hemorrhage	3-5-6	3+1	7	Brain tissue around right middle cerebral artery aneurysm	Poor
	2	65	Male	Aneurysms rupture with subarachnoid hemorrhage	4-5-6	1	1	Brain tissue around right middle cerebral artery aneurysm	Good
	3	57	Female	Aneurysms rupture with subarachnoid hemorrhage	4-5-6	1	7	Brain tissue around anterior communicating artery	Good

Supplemental Table 2. Statistical table

	Description	Hours/days after SAH or sham surgery or OxyHb treatment	In vivo or vitro	Test used	Stat-value	One- or two-tailed P value
Fig 2c	Relative protein	3h, 6h, 12h, 24h,	in vivo	Ordinary	F(6,35)=5.609, P<0.001;	Two-tailed

	level of CX3CL1	72h, 1w		one-way ANOVA	Bonferroni's post hoc test, P=0.0078 (6h vs sham), P=0.0014 (12h vs sham); η^2=0.4902	
Fig 2d	Relative protein level of CX3CR1	3h, 6h, 12h, 24h, 72h, 1w	in vivo	Ordinary one-way ANOVA	F(6,34)=7.249, P<0.001; Bonferroni's post hoc test, P=0.0183 (6h vs sham), P=0.0174 (12h vs sham); η^2=0.5019	Two-tailed
Fig 3c	Relative protein level of CX3CL1	12h	in vivo	Ordinary one-way ANOVA	F(3,20)=15.97, P<0.001; Tukey's post hoc test, P<0.001 (SAH vs sham), P=0.0054 (OE vs Vector); η^2=0.7055	Two-tailed
Fig 3d	Relative protein level of CX3CR1	12h	in vivo	Ordinary one-way ANOVA	F(3,20)=9.868, P<0.001; Tukey's post hoc test, P=0.0085 (SAH vs sham), P=0.0043 (OE vs Vector); η^2=0.5967	Two-tailed
Fig 3f	FJC	12h	in vivo	Ordinary one-way ANOVA	F(3,20)=136.3, P<0.001; Tukey's post hoc test, P<0.001 (SAH vs sham), P<0.001 (OE vs Vector); η^2=0.9112	Two-tailed
Fig 4a	Neurobehavioral scores	24h	in vivo	Mann-Whitney U test	P<0.001 (SAH vs sham), P<0.001 (OE vs Vector)	Two-tailed
Fig 4b	Adhesive-removal time	1d, 3d, 7d, 14d	in vivo	Two -way ANOVA	F(3,36)=66.29, P<0.001; Tukey's post hoc test, P<0.001 (SAH vs sham), P=0.0027 (OE vs Vector)； Bonferroni's post hoc test, P<0.001 (1d: OE vs Vector); η^2=0.6866	Two-tailed
Fig 4c	Rotarod test	1d, 3d, 7d, 14d	in vivo	Two -way ANOVA	F(3,36)=153, P<0.001; Tukey's post hoc test, P<0.001 (SAH vs sham), P<0.001 (OE vs Vector); Bonferroni's post hoc test, P=0.0048 (3d: OE vs Vector); η^2=0.7270	Two-tailed

Fig 4d	Escape latency	22d-26d	in vivo	Two -way ANOVA	F(3,20)=59.54, P<0.001; Tukey's post hoc test, P<0.001 (SAH vs sham), P<0.001 (OE vs Vector); η^2=0.7899	Two-tailed
Fig 5b	Relative protein level of CD45	12h	in vivo	Ordinary one-way ANOVA	F(3,20)=15.77, P<0.001; Tukey's post hoc test, P<0.001 (SAH vs sham), P=0.0015 (OE vs Vector); η^2=0.7029	Two-tailed
Fig 5c	Relative protein level of MHC Class II	12h	in vivo	Ordinary one-way ANOVA	F(3,20)=13.44, P<0.001; Tukey's post hoc test, P=0.0024 (SAH vs sham), P=0.0011 (OE vs Vector); η^2=0.6687	Two-tailed
Fig 5d	Relative protein level of C/EBPα	12h	in vivo	Ordinary one-way ANOVA	F(3,20)=8.989, P<0.001; Tukey's post hoc test, P=0.0075 (SAH vs sham), P=0.0081 (OE vs Vector); η^2=0.5742	Two-tailed
Fig 5g	Relative protein level of MCPIP	12h	in vivo	Ordinary one-way ANOVA	F(3,20)=8.37, P<0.001; Tukey's post hoc test, P=0.0031 (SAH vs sham), P=0.0482 (OE vs Vector); η^2=0.5566	Two-tailed
Fig 5h	Relative protein level of Runx1	12h	in vivo	Ordinary one-way ANOVA	F(3,20)=9.627, P<0.001; Tukey's post hoc test, P=0.0037 (SAH vs sham), P=0.0088 (OE vs Vector); η^2=0.5911	Two-tailed
Fig 6a	Relative level of TNF-α	12h	in vivo	Ordinary one-way ANOVA	F(3,20)=75.18, P<0.001; Tukey's post hoc test, P<0.001 (SAH vs sham), P<0.001 (OE vs Vector); η^2=0.9185	Two-tailed
Fig 6b	Relative level of IL-1α	12h	in vivo	Ordinary one-way ANOVA	F(3,20)=91.55, P<0.001; Tukey's post hoc test, P<0.001 (SAH vs sham), P<0.001 (OE vs Vector); η^2=0.8985	Two-tailed
Fig 6c	Relative level of C1q	12h	in vivo	Ordinary one-way	F(3,20)=46.58, P<0.001; Tukey's post hoc test, P<0.001 (SAH vs	Two-tailed

Fig 7d	Relative level of 12h exosomal miR124	in vitro	ANOVA Ordinary one-way ANOVA	sham), P<0.001 (OE vs Vector); η^2=0.8527 F(5,12)=93.1, P<0.001; Tukey's post hoc test, P<0.001 (Control+GW4869 vs Control+Vehicle), P<0.001 (OxyHb vs Control), P<0.001 (OxyHb +Vehicle vs Control+Vehicle), P<0.001 (OxyHb+GW4869 vs OxyHb +Vehicle); η^2=0.9749	Two-tailed

Supplemental Table 3. Modeling situation.

Groups	Mortality Rate	Excluded
**Experiment 1**		
sham	0% (0/12)	0
SAH (3h,6h,12h,24h,72h,1w)	15.6% (14/90)	4
**Experiment 2**		
sham	0% (0/12)	0
SAH	12.5% (2/16)	2
SAH+Vector	25% (4/16)	0
SAH+OE	22.2% (4/18)	2
**Experiment 3**		
sham	0% (0/10)	0
SAH	21.4% (3/14)	1
SAH+Vector	14.3% (2/14)	2
SAH+OE	15.4% (2/13)	1
**Total**		
sham	0% (0/34)	0

SAH	17.1% (29/181)	12
